# Deceased

**Published:** 1884-11

**Authors:** 


### DECEASED.

*The New England Journal of Dentistry* is no more. Its editors and publishers, probably feeling that there is small encouragement to labor for others, propose to look after their personal interests for a time. For nearly three years they have wrought faithfully for their profession, and, it is needless to say, without other reward than that which a satisfied conscience bestows. The Journal has done some excellent work. But it has been pecked at by daws, hawked at by mousing owls, and the self-sacrificing and disinterested labors of its editors have been mocked where they should have received highest honor. Perhaps the Journal has not always delved with discretion, and it may have wrought hard to establish a fallacy; but its work has been unselfish, and prompted by noble motives. It may at times have offended some, but what positive man or journal, worthy the attention of any earnest and conscientious laborer, has not done so? Who values the victories of My Uncle Toby in the back garden? It is he who gives as well as receives sturdy blows, that is worthy of honor. Another independent dental journal has succumbed. Disinterested pens are dried, and self-denying labors have ceased. There is rest for weary brains, but — is it well for the profession?

